# Precautions in the Use of Salicylic Acid

**Published:** 1877-01

**Authors:** 


Precautions in the Use of Salicylic Acid.—After relating some cases of rheumatism in most of which the acid had been very useful, and one in which it had not, and after noting its effect in reducing the temperature, and in one case, he says, of producing intermittent pulse, the author proceeds:

These cases, although perhaps too few to serve as a basis to any positive conclusions, lead to the belief that in acute rheumatism of adults salicylic acid should be administered in doses of five grains every three hours, for two days; and then, if the effect is not apparent, five grains every two hours, and even every hour for another forty-eight hours at the most, unless vomiting first supervenes. If, however, on the second, third, or fourth day profuse perspiration comes on, with reduction of pulse and heat of skin, the temperature will have to be accurately observed at intervals of six or eight hours; and when it comes down to 99 F., the remedy will have to be suspended and quinine given instead; and if the thermometer marks a lowering below the normal, with brandy and carbonate of ammonia freely to sustain the vital powers. With such precautions, treating the case, so to speak, *with thermometer in hand*, we shall probably be able to avoid severe and dangerous prostration, which in a weak patient, and in cases seriously complicated, might be followed by fatal results.

It appears obvious that the remarkable power this non-poisonous antiseptic seems to possess of controlling rheumatic fever, will bring, if it shall be proved that it exists, great support to that theory of the origin of certain diseases which my observations and my experiments induce me to embrace.—*Richardson, from Germ. di Med. Mil.—Gaz. Med. Italiana.*

